# Systemic Compensatory Response to Neonatal Estradiol Exposure Does Not Prevent Depletion of the Oocyte Pool in the Rat

**DOI:** 10.1371/journal.pone.0082175

**Published:** 2013-12-16

**Authors:** Clémentine Chalmey, Franck Giton, Frédéric Chalmel, Jean Fiet, Bernard Jégou, Séverine Mazaud-Guittot

**Affiliations:** 1 Institut National de la Santé et de la Recherche Médicale, Unité 1085 Institut de Recherche en Santé Environnement et Travail, Institut Fédératif de Recherche 140, Université de Rennes 1, Rennes, France; 2 AP-HP, Hôpital H. Mondor - A. Chenevier, service de Biochimie et de Génétique, Créteil, France; 3 Institut National de la Santé et de la Recherche Médicale, U955 Équipe 07, Créteil, France; 4 Ecole des Hautes Études en Santé Publique, Rennes, France; Michigan State University, United States of America

## Abstract

The formation of ovarian follicles is a finely tuned process that takes place within a narrow time-window in rodents. Multiple factors and pathways have been proposed to contribute to the mechanisms triggering this process but the role of endocrine factors, especially estrogens, remains elusive. It is currently hypothesized that removal from the maternal hormonal environment permits follicle formation at birth. However, experimentally-induced maintenance of high 17β-estradiol (E2) levels leads to subtle, distinct, immediate effects on follicle formation and oocyte survival depending on the species and dose. In this study, we examined the immediate effects of neonatal E2 exposure from post-natal day (PND) 0 to PND2 on the whole organism and on ovarian follicle formation in rats. Measurements of plasma E2, estrone and their sulfate conjugates after E2 exposure showed that neonatal female rats rapidly acquire the capability to metabolize and clear excessive E2 levels. Concomitant modifications to the mRNA content of genes encoding selected E2 metabolism enzymes in the liver and the ovary in response to E2 exposure indicate that E2 may modify the neonatal maturation of these organs. In the liver, E2 treatment was associated with lower acquisition of the capability to metabolize E2. In the ovary, E2 depleted the oocyte pool in a dose dependent manner by PND3. In 10 µg/day E2-treated ovaries, apoptotic oocytes were observed in newly formed follicles in addition to areas of ovarian cord remodeling. At PND6, follicles without any visible oocyte were present and multi-oocyte follicles were not observed. Our study reveals a major species-difference. Indeed, neonatal exposure to E2 depletes the oocyte pool in the rat ovary, whereas in the mouse it is well known to increase oocyte survival.

## Introduction

Mammalian birth is characterized by dramatic endocrine changes and, in female rodents, this occurs concomitantly with crucial morphogenetic processes in the ovaries. The formation of ovarian follicles that occurs in the days following birth, will determine the whole reproductive life of the female. Although a fetus is likely to be exposed to high levels of steroids (in particular progesterone and estradiol [1], [2]), the maternal liver and excretory organs manage their biotransformation and elimination. The fetus is also protected by the placental barrier and by binding proteins. Circulating steroids undergo a rapid decrease in the days following birth and reach nadir at the end of the first week until the newborn synthesizes its own steroids at the beginning of the infantile period [1]–[6]. This sudden release from maternal hormonal impregnation is associated with a rapid increase in the expression and activity of enzymes involved in hormone metabolism and detoxification machinery in the newborn liver [7], [8]. As a consequence, the newborn acquires the capability to metabolize a variety of hormones and xenobiotics during the first week of life.

In the ovary, the formation of functional units (the follicles) from the immature fetal ovarian cords also takes place within the three days following birth [9], [10]. Ovarian cords are composed of clusters of germ cells progressing through the first prophase of meiosis, surrounded by pregranulosa cells, and delineated by a continuous basal membrane. They fragment thanks to the deposition of a new basement membrane [11]. This fragmentation is associated with the separation of oocytes, which remained interconnected as nests or cysts during synchronous mitosis [9], [10], and with a massive wave of degeneration specifically targeting oocytes resulting from both apoptotic and autophagic mechanisms [12]–[15]. A number of factors, mechanisms and pathways have been suggested to be involved in follicle formation. These include the endogenous meiosis clock, central signals and neurotrophic factor signaling, growth factor signaling (especially those of the transforming growth factor family) and transcription factors (for review, [10]). In addition, several studies have highlighted endocrine factors such as progesterone and 17β-estradiol (E2) as candidate actors of follicle histogenesis in several specie from mice, rats to cattle and primates [4], [16]–[18]. It is currently hypothesized that the decrease in maternal E2 impregnation that follows birth permits oocyte cyst breakdown, and consequently, follicle formation. Therefore, maintenance of high E2 levels or exposure to E2-mimicking molecules may result in inhibition of oocyte cyst breakdown. However, experimentally-induced high neonatal E2 levels have subtly-diverging immediate effects depending on the species (and even strains) in terms of oocyte survival and ovarian cord fragmentation, *i.e.* follicle formation. High post-natal E2 levels increase oocyte survival but inhibit follicle formation in mice [4], [18]. However, they promote both follicle formation and oocyte survival in the hamster [19], [20]. In the baboon, maintenance of maternal E2 levels are required for follicle formation since a treatment with an aromatase inhibitor leads to a sharp inhibition of the process [21]. In the rat, E2 moderately inhibits follicle assembly but increases primordial-to-primary follicle transition *in vitro* and leads to hypoplastic ovaries from post-natal day (PND) 4 onward [16], [22]. A typical feature of neonatal exposure to E2 or xeno-estrogens such as diethylstilbestrol (DES), bisphenol A (BPA) and genistein (GEN) is a high incidence of multi-oocyte follicles (MOFs) in peripubertal mice and rats [18], [23]–[28]. Although these MOFs are thought to arise from reduced oocyte cysts breakdown, oocyte apoptosis and/or basement membrane neosynthesis and deposition, the fine etiology of this morphogenetic abnormality and the role of estrogenic compounds are still obscure.

Increasing concerns of xeno-estrogens impact on health have led to experimental studies using compounds displaying various estrogenic activities (DES, BPA, GEN …). The use of E2 or estradiol-like molecules with distinct kinetic properties (including ethinyl estradiol, estradiol-benzoate, estradiol–valerate and estradiol-cypionate) as reference molecules has increased the complexity of this issue. Although E2 is widely used as a reference molecule in comparisons with xeno-estrogens, its own mechanism of action is not completely understood. In addition, due to the availability of transgenic mice, many studies addressing the effects of a neonatal exposure to E2 and xeno-estrogens on follicle formation have been carried out using mice. Although the rat has often been the model of choice to study molecules affecting the sexual differentiation of the central nervous system [29], [30], the direct effects of neonatal E2 exposure to the ovary remains unclear in this species.

As a consequence, the aim of this study was to better clarify the immediate adverse effects of E2 on the ovary in the rat. To that purpose, we injected newborn pups with E2 during the short time window corresponding to follicle formation. As a prerequisite for an acute evaluation of E2 exposure and in an attempt to define effective doses, we examined dose-effects using doses between 10 µg/day/animal and 0.01 µg/day/animal and compared the dynamics of the resulting plasma E2 in the pups. To gain insights into the fate of E2, we also evaluated dynamics of circulating metabolites and the effects of E2 treatment on the expression of the enzymes of the detoxification machinery in the newborn liver. In the ovary, we searched for detoxification capabilities and evaluated the impact of E2 exposure on: i) these possible local metabolizing capabilities, ii) oocyte survival and iii) follicle formation.

## Materials and Methods

### Ethic Statement

The animal facility is licensed by the French Ministry of Agriculture (agreement # C 35-238-19). All the experimental procedures followed ethical principles according to the NIH Guide for Care and Use of the Laboratory Animals and were approved by the Rennes Animal Experimentation Ethics Committee (#R-2012-CCh-01).

### Animal Handling and Treatments

Adult Sprague-Dawley rats were conventionally housed at adequate temperature (22±5°C) and humidity (around 55%) with 12∶12 h light/dark cycles and free access to food and tap water. Their food contained soy (Special Diets Services, Witham, England). Females were mated overnight with males. The following day was considered to be embryonic day 0 (e0). Pregnant females were isolated three days before birth, which generally occurs at e22.5. Newborn females received a subcutaneous injection of 17β-estradiol or corn oil (vehicle–negative control) (Sigma Aldrich) within a few hours after birth (PND0) and on two subsequent days (PND1 and 2). Animals were divided in five groups: one group received the negative control and four experimental groups received 0.01, 0.1, 1 or 10 µg/day of 17β-estradiol.

### Organs and Plasma Collection

Animals were killed by decapitation in the morning of PND0, 1, 2, 3 or 6. Trunk blood was collected into heparinized tubes and centrifuged, and plasma was kept frozen (−20°C) until steroid hormone assay. Ovaries were collected at each age, separated from the ovarian capsule and fixed overnight in Bouin’s solution for immunohistochemistry or in 2% paraformaldehyde-PBS (pH 7.2) for 1 h at 4°C for *in situ* hybridization. Livers and ovaries were removed and snap frozen for RNA extraction.

### Hormone Measurements

Serum samples from two or three animals were pooled and randomized before assaying. Estrone (E1), estradiol (E2), estrone-sulfate (E1-S) and estradiol-sulfate (E2-S) were assayed in two steps using mass spectrometry coupled with gas chromatography [31], [32]. Sera were overloaded with deuterated steroid internal standards (CDN isotopes Inc., Canada) and extracted with 1-chlorobutane. The organic extracts were purified on conditioned LC-Si SPE columns (Varian, Les Ulis, France). E1 and E2 were derivatized with pentafluorobenzoyl chloride (103772-1G, Aldrich, Steinheim, Germany). The final extracts were reconstituted in isooctane and transferred into conical vials for injection into the GC system (6890N, Agilent Technologies, Massy, France) using 50% phenylmethylpolysiloxane VF-17MS capillary columns (20 m×0.15 mm, internal diameter, 0.15 µm film thickness) (Varian, Les Ulis, France). An HP5973 (Agilent Technologies, Massy, France) quadrupole mass spectrometer equipped with a chemical ionization source and operating in single ion monitoring (SIM) mode was used for detection. E1-S and E2-S retained in the aqueous phase of the extraction were measured after acid solvolysis as E1 and E2, respectively [33]. Accuracy, target ions, corresponding deuterated internal control, range of detection, low limit of quantification (LLOQ), and intra & inter assay CVs of the quality control serums are reported in table S1.

### *In situ* Hybridization and Coupled Immunofluorescence

Fixed ovaries were cryoprotected in 20% (wt/vol) sucrose in PBS for 1 h, embedded in Tissue-Tek OCT compound (Miles, Inc., Elkhart, IN), cut into 8 µm-thick sections, mounted onto 3-aminopropyltriethoxysilane-treated glass slides (Sigma-Aldrich Corp., St. Louis, MO) and stored at −20°C. The cDNAs used for synthesis of the different riboprobes were obtained by RT-PCR and subcloned into pGEMT_easy_ (Promega). The nucleotide sequences were verified by sequencing. Primers and product lengths are given in Table 1. Riboprobes were generated by transcription with digoxigenin-labeled deoxy-UTP and the appropriate SP6 or T7 RNA polymerase (Roche). *In situ* hybridization on frozen sections was carried out as previously described [5]. Detection of apoptotic cells was performed on sections previously treated for Ybx2 *in situ* hybridization using the *in situ* cell death detection kit, Fluorescein (terminal deoxynucleotidyltransferase-mediated deoxy-uridine 5′-triphosphate-fluorescein nick end labeling (TUNEL), Roche). After PBS washing, sections were incubated with the TUNEL reaction mixture containing terminal transferase for 1 h at 37°C. For double labeling of sections (if appropriate after detection of Ybx2 or Gper mRNA by *in situ* hybridization), sections were rinsed in PBS and incubated overnight at 4°C with Esr2 (Biogenex, AR385-5R, RTU), fibronectin (Sigma Aldrich, F3648, 1∶100) or Ybx2 (Santa Cruz Biotechnologies, Inc. Santa Cruz, CA, sc-21316, 1∶200) antibodies. An antigen retrieval procedure was necessary before Esr2 antibody incubation (10 mM citrate buffer, pH 6, at 80°C for 45 min). After washing, sections were incubated with secondary 488- or 594-Alexa anti-rabbit (Esr2, fibronectin) or anti-goat (Ybx2) antibodies (IgG Alexa antibodies, Invitrogen, 1∶500). The second primary antibody was incubated overnight with the sections at 4°C, and the second antigen/antibody complex was then reacted with the appropriate secondary antibody. For TUNEL associated with fibronectin detection, the TUNEL reaction was performed as described and followed by fibronectin labeling. Fluorochrome-labeled sections were mounted in Vectashield® containing the DNA stain DAPI (Vector Laboratories), and analyzed with a Zeiss Axio Imager M1 fluorescence microscope connected to a digital camera (Carl Zeiss New York, NY).

**Table 1:** Primers used for qPCR and *in situ* hybridization (*).

Gene	Upstream primer	Downstream primer	Product length (bp)	Annealing T°C
Bax	CTAGCAAACTGGTGCTCAAGG	GGAGGAAGTCCAGTGTCCAG	84	60
Bcl2	AACATCGCTCTGTGGATGACT	ACAGCCAGGAGAAATCAAACA	133	60
Cyp1b1	GCAGCCGCCTTCCTGGTAGC	CCACGCGCCCTGTCCCTACT	116	60
Cyp2b1/2	ATGTTTGGTGGAGGAACTGC	CTGGCGGTCTGTGTAGTCAA	130	63
Eif4e	AGCAATATGGACGACTGAATGTGA	TGTCTGCGTGGGACTGATAACC	119	60
Esr1	GATCAAGTTCACCTTCTGGA	AGCAAGTTAGGAGCAAACAG	107	60
Esr1 *	CTACAGGTCCAATTCTGACA	TGGAGACATGTAGTCATTATG	220	58
Esr2	GAAGCTGAACCACCCAATGT	CAGTCCCACCATTAGCACCT	210	60
Esr2 *	ACGGTGGGCATGCACCCC	GCCAATCATGTGCACCAG	201	58
Figla	ATGGACACGTCGTCGCCTGC	TGGCCACCATACGCCCAAGG	524	60
Foxa3	ATGCTGGGCTCAGTGAAGAT	GGGAGAGCTAAGAGGGTTCAA	147	60
Gper	TCTACCTAGGTCCCGTGTGG	AAGCTCATCCAGGTGAGGAA	418	60
Gper *	TCTACCTAGGTCCCGTGTGG	AAGCTCATCCAGGTGAGGAA	418	58
Gsta2	GGCAAAAGACAGGACCAAAA	GGCTGCAGGAACTTCTTCAC	231	62
Gstm5	GGTTTGCAGGAGAAAAGCTG	TGATTGGCATCTTGAAGCAG	186	62
Gstp1	GGGCATCTGAAACCTTTTGA	GAGCCACATAGGCAGAGAGC	175	63
Hnrnpk	TGCTGATGAAACTTGGGACTCTG	CGAATTTGTTTAATCCGCTGACC	222	60
Hsd17b2	GACAAAGGACTGTGGGCTGT	AACACCTTGGTGACCTCGAC	137	60
Nobox	GAAGACATGGGACCTCAGGA	GCCGAAAGGAAATGAAAACA	192	60
Nr1i2	GACGGCAGCATCTGGAACTAC	TGATGACGCCCTTGAACATG	112	60
Rbp4	GGACGAGTCCGTCTTCTGAG	AAAGGAGGCTACACCCCAGT	114	60
Scp1	AGCTTTTGGGAGAGGTTGAGAA	TCAGCTATTTTATGTTGGCATCGT	97	62
Snx17	CCTCTACCCAAGAGGAGATTTATACAA	CCAAGAGGGAACAGAACAAGT	100	60
Sult1e1	GTGGAAAAATGCAAGGAGGA	GCTTAGCTGGCAGGTGAGTT	139	58
Ugt1a1	ACACCGGAACTAGACCATCG	TTGGAACCCCATTGCATATT	153	62
Ybx2 *	CACCTCCCTTCTTCTATCGA	GGTGATGCCTCTGAACAATA	637	58

### Stereological Counts

For oocyte counts, PND0, 1, 2 and 3 ovaries were fixed with Bouin’s solution, dehydrated and paraffin embedded according to standard procedures. One out of every five 5 µm sections were mounted with albumin on TESPA-treated glass slides. After dewaxing and rehydration, an antigen retrieval procedure was performed with citrate buffer 10 mM pH 6 at 80°C for 45 min. Unspecific sites were saturated with 10% Bovine Serum Albumin (BSA)-PBS and the sections were incubated overnight at 4°C with the primary antibody (Gena, clone TRA98, Clinisciences, 1∶100 diluted in Dako antibody diluent). After washes in PBS, sections were reacted with a biotinylated secondary antibody (Sigma Aldrich; 1∶500 in PBS) and incubated for 4h at room temperature. An avidin-biotin complex linked to a peroxidase (Vectastain Elite ABC kit, Vector Labs) was used to bind to diaminobenzidine, a peroxidase substrate (Sigma Aldrich). Sections were counterstained with Masson’s Hemalun, dehydrated and mounted in Eukitt (Kindler GmbH). For total oocyte counts, Gena/TRA98-positive cells were counted by using the Computer-Assisted Stereology Toolbox (CAST) Grid System (Olympus, Copenhagen, Denmark) on a light microscope (Olympus BX S1). At first, we delineated the ovarian borders at low magnification. The ovarian volume was deducted by multiplying the sum of the surfaces of each section by the thickness of the sections (5 µm) and another time by 5 because measurement was done every fifth sections. Then, we counted the immunopositive cells using the oocyte nucleus as a marker at 100X using a high-numerical-aperture objective lens on each section. All oocytes were added together to reflect an estimation of the total oocyte number per ovary. For dying oocyte counts, apoptotic cells were detected by the TUNEL technique using the Apoptag cell kit (Millipore, S1700) according to manufacturer’s instructions. TUNEL-positive oocytes were counted in every fifth section and added together to give the total apoptotic oocytes per ovary. Stained sections were examined under a light microscope (Olympus BX51).

### RNA Extraction and Polymerase Chain Reaction (PCR)

RNA extractions from ovaries and livers were carried out with a Nucleospin XS kit (Macherey Nagel; 740902) according to manufacturer’s instructions. Total RNAs (250 ng) were reverse transcribed with random primers and Moloney Murine Leukemia Virus Reverse Transcriptase (Invitrogen). Conventional PCR was performed using Taq polymerase (Qiagen) in a Peltier thermocycler (MJ Research, Bio-Rad DNA engine) using the primers listed in Table 1. PCR consisted of an initial denaturation at 94°C for 3 min, 35 (or 42 in the case of Sult1e1) cycles of 94°C for 30 s, 1 min of annealing, 72°C for 30 s and a 10 min 72°C final extension. Quantitative PCR was performed using the GoTaq© Master mix (Promega) according to manufacturer’s instructions with 0.5 µl cDNA template in an Applied Biosystems 7500 Real-Time PCR system. The following amplification program was used: a 2 min holding stage at 50°C, initial denaturation of 10 min at 95°C, 40 cycles of 1 sec amplification at 95°C and 1 min at 60°C for annealing and extension. Dissociation curves were produced using a thermal melting profile performed after the last PCR cycle. To avoid amplification of contaminating genomic DNA, primer pairs were selected on either side of an intron. Snx17 mRNA was used as an internal control for normalization. Results were expressed by ΔΔCT method as *n*-fold differences in target gene expression, relative to the reference gene and calibrator sample which is constituted of an equal mixture of all the samples tested specifically in each tested organ.

### Data Analysis

Microarray data analysis was carried out on datasets from Kezele *et al,* [34], GSE 9300 (GSM237013-237016) using the Annotation, Mapping, Expression and Network (AMEN) analysis software [35]. GeneChip data were normalized using the Robust Multi-Array Average (RMA) method and the resulting expression values for ovary replicates at PND0 and PND4 were averaged. Probe sets yielding a signal higher than the detection threshold (median of the normalized dataset, cutoff 7.47), and a fold-change ≥1.5 between PND0 and PND4 averaged ovary samples, were selected. A Linear Model for Microarray Data (LIMMA) statistical test (F-value adjusted with the False Discovery Rate method: p≤0.05) was used to identify probe sets with significantly different expression. For further classification, we divided the selected probe sets into groups being significantly decreased or increased in PND4 ovary samples as compared to PND0. We restricted our subsequent analysis to probe sets corresponding to genes associated with the “steroid metabolic process” Gene Ontology annotation term (GO:0008202).

### Statistical Analysis

Quantitative PCR data and cell counts are presented as mean ± SEM. Blood and ovaries for quantitative PCR from different animals were pooled to form one sample and several pools from different litters were used for a given treatment and time point. The sizes of the pools are given in each figure legend. For oocyte counts, 4–13 ovaries were used, as specified for each parameter in the corresponding figure legend. An analysis of the variance (one-way or two-ways ANOVA) followed by the appropriate post-hoc test was used to compare differences between groups, as specified in each figure legend. Significance was accepted at a confidence level of p≤0.05. Statistical analyses were performed using the SigmaStat 2.0 software package (Systat Software Inc, San Jose, CA).

## Results

### Response of the Whole Organism to E2 Exposure

**Figure 1:**
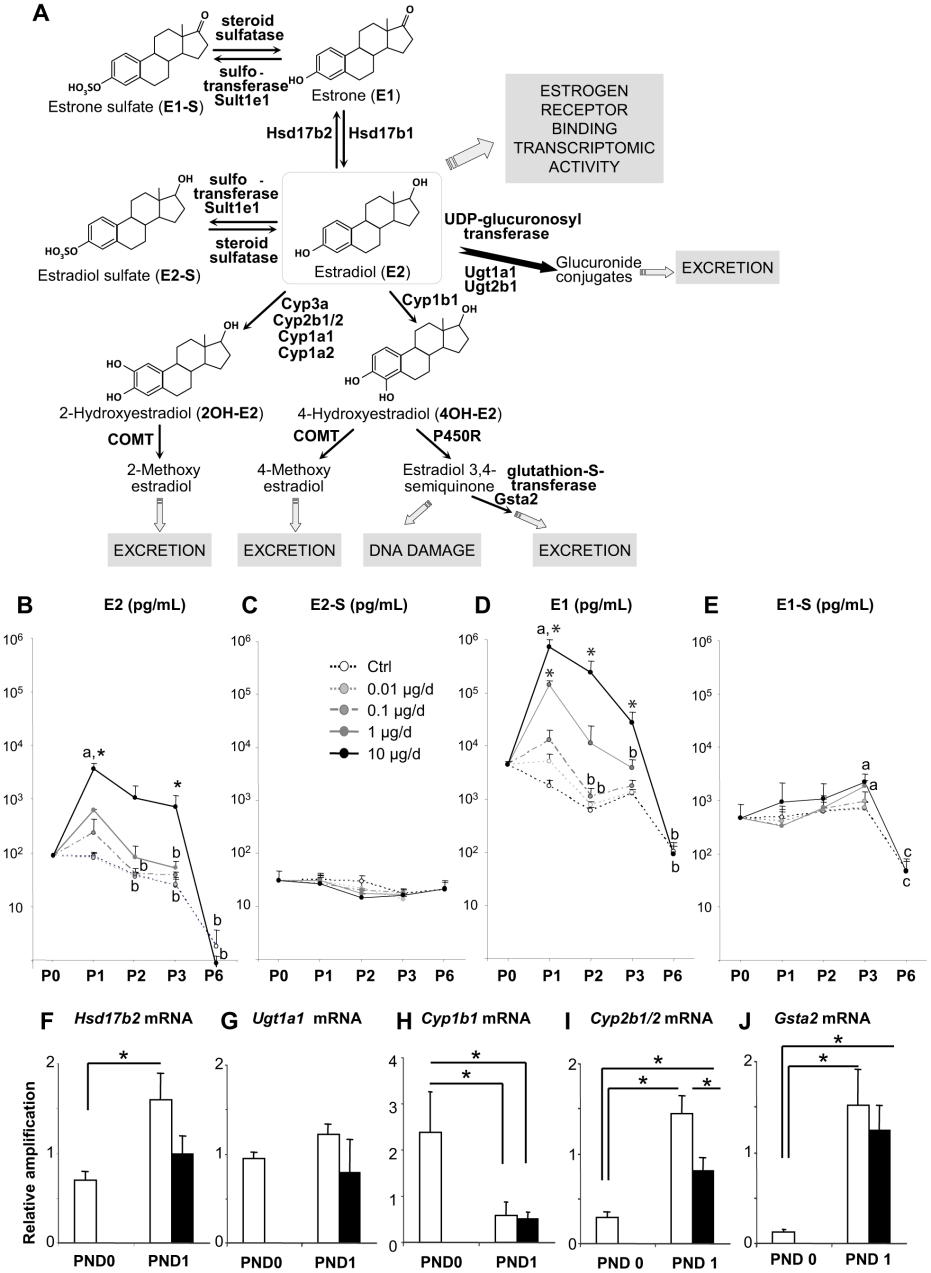
Plasma and hepatic reaction to acute estrogen exposure. A. Representative scheme of E2 biological activity and detoxification pathways. E2 can go through oxidative metabolism and be converted in E1 by 17bHsd2 or in hydroxyl-metabolites by enzymes of the Cyp family. Then it can be methylated by COMT, reduced by P450 reductase (resulting in DNA damage) or excreted after conjugative metabolism by GST. E2 can also be directly sulfo-conjugated by SULT or gluco-conjugated by UGT, and excreted. B–E. Plasma estradiol (B) and derivatives E2-sulfate (E2-S; C), estrone (D) and estrone-sulfate (E1-S; E) were measured by GC/MS from the day of birth (P0) to PND6 (P6). Each point represents the mean ± SEM of at least four pools of two animals each. A two-way ANOVA indicated that there was a significantly different profile for the 10 µg/d E2 treatment than for all other treated or control groups (p<0.001), E1 (p<0.001), E1-S (p = 0.002). Hormonal levels varied according to time, independent of treatment, for each metabolite tested: E1 (p<0.001), E2 (p<0.001), E1-S (p<0.001) and E2-S (p<0.001) (n = 4–5 pools of 3 to 4 animals at PND0, 4–5 pools of 2–4 animals at PND1, 4 pools of 2–3 animals at PND2, 4 pools of 2 animals at PND3 and 8 animals at PND6). F–J. Quantitative RT-PCR for *Hsd17b2* (F), *Ugt1a1* (G), *Cyp1b1* (H), *Cyp2b1/2* (I) and *Gsta2* (J) using liver samples of controls (white bars) and animals treated with 10 µg E2 at PND0 and PND1 (black bars) shows E2 impairment on post-natal *Hsd17b2*, *Cyp2b1/2* and *Gsta2* expression dynamics. Each bar represents mean ± SEM of the fold-change in target gene expression relative to a reference gene Snx17 and calibrator sample. Each point represents mRNA from 3 pools of ovaries from 3 animals. *p<0.05 (two-way ANOVA, followed by Tukey test). **a** shows an increase from PND0, **b** shows a decrease from PND1, **c** shows a decrease from PND3 and * shows a difference from the age-matched control group.

Besides its biological activity involving by binding to receptors and subsequent transcriptomic activity, E2 is transformed into several possible metabolites before excretion (Fig. 1A). To monitor the dynamics of plasma E2 levels after E2 treatment in comparison to physiological levels, we measured E2 levels in rat neonates at PND0 (before injections) and 16 h after each injection on days PND1 to PND3 and PND6 (Fig. 1B). The mean E2 levels significantly decreased after birth in control females and drastically dropped at PND6. All treated groups followed the same trend, except the 0.01 µg group which did not differ from controls. E2 plasma levels increased at PND1 following the first injection of E2 (statistically significant in only the 10 µg/d-treated group), but significantly decreased until PND3 in spite of repeated injections. Plasma E2 levels of the 10 µg/d-treated group did not return to baseline at PND3 but were the same as control levels from PND6 onwards.

To explore the fate of E2 in the females, we quantified the presence of several major E2 metabolites (estradiol-sulfate (E2-S), estrone (E1), and estrone-sulfate (E1-S)), at the same time points (Fig. 1B–E). Estriol (E3), another natural E2 metabolite, was also assayed but all the samples displayed concentrations below the detection limit (10 pg/ml). In contrast to E2, E2-S remained constant in all samples, irrespective of the huge raise in E2 levels in the 10 µg/d group (Fig. 1C). Time, but not treatment, had a significant effect on E2-S level (P<0.001). E1 profiles were highly similar to those of E2 in accordance with the injected dose (Fig. 1D). The higher E1 levels at PND1 were statistically significant in the 10 µg/d and 1 µg/d groups. Similar to E2, E1 levels dramatically dropped at PND6 with time and treatment having independent effects. E1-S peaked at PND3, and this increase was significant in the 10 µg/d and 1 µg/d groups. In all groups, plasma E1-S levels decreased between PND3 to PND6 (Fig. 1E). The two-way analysis of variance revealed that the variation of all metabolite levels was statistically correlated with time, independently of treatment. The dose of injected E2 significantly affected the mean values of all metabolites except E2-S. The multiple comparison procedure identified the E2 10 µg/d group as different.

The increase in E1 levels after E2 injections suggested a metabolic conversion between those two forms. We therefore investigated variation in the expression of *Hsd17b2*, and of several phase-I and phase-II enzymes involved in hepatic detoxification of estrogens (*Ugt1a1, Cyp1b1, Cyp2b1/2* and *Gsta2*), in response to 10 µg/d E2 by quantitative PCR in the liver (Fig. 1F–J). mRNA levels of *Hsd17b2* and *Cyp2b1/2* increased from PND0 to PND1 in the control group but there was no variation in the E2-treated group (Fig. 1F, I). *Ugt1a1* levels did not vary significantly with age or treatment (Fig. 1G). The levels of *Cyp1b1* mRNA (Fig. 1H) were lower at PND1 than at PND0 and treatment had no effect. In contrast, the levels of *Gsta2* mRNA were higher at PND1 than at PND0 in 10 µg/d-treated and control animals (Fig. 1J).

### Ovarian Estrogen Receptivity in Response to E2 Exposure

**Figure 2:**
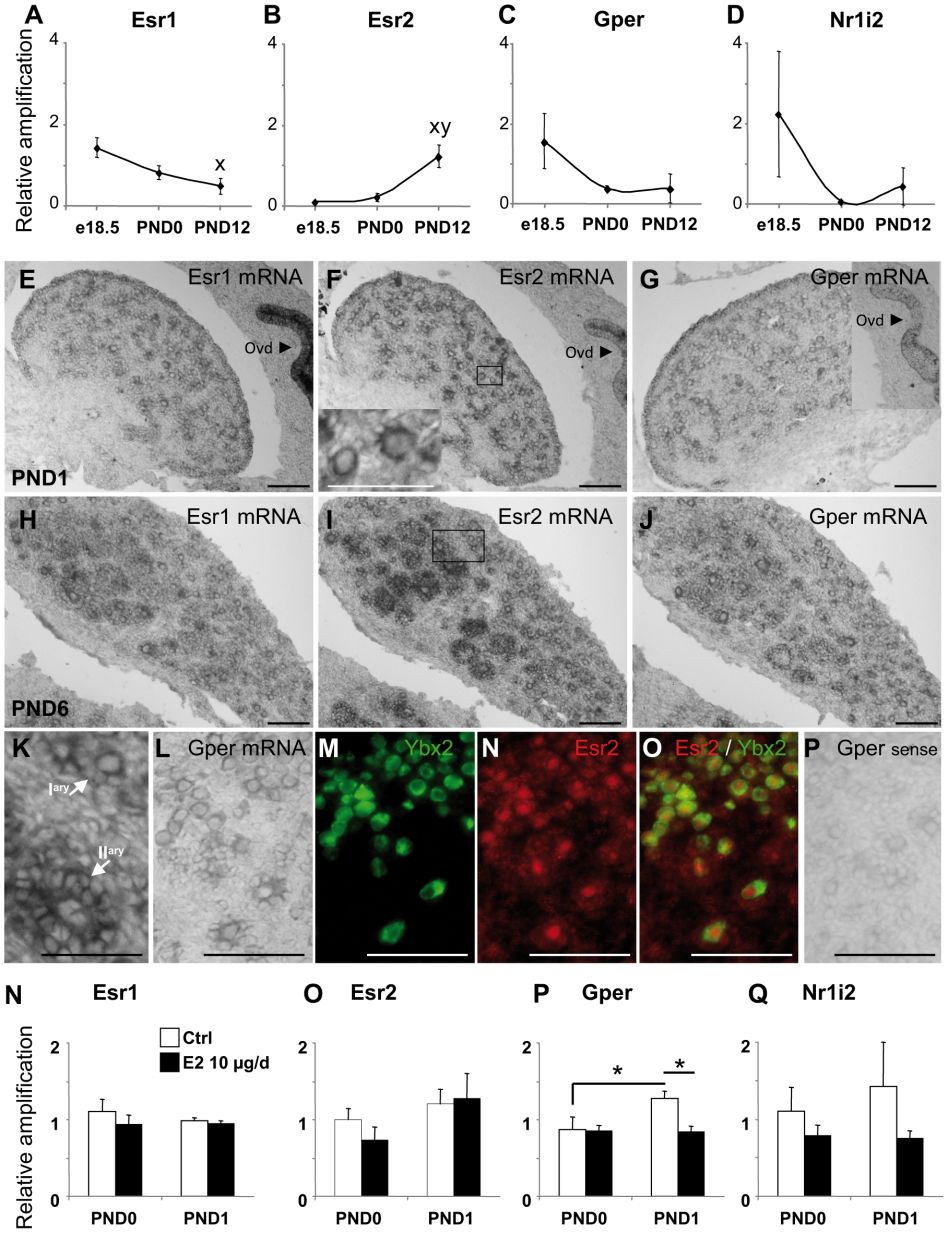
Perinatal ovarian receptivity to estrogens. A–D: Quantitative RT-PCR for Esr1 (A), Esr2 (B), Gper (C) and Nr1i2 (D) performed on control ovaries at e18.5, day of birth (PND0) and PND12. Each point is constituted by three pools of at least four animals. Data points represent the mean ± SEM of the fold-change in target gene expression relative to a Snx17 reference gene and calibrator sample. Each point represents mRNA from 3 pools of ovaries from 3 animals. *p<0.05 (ANOVA, followed by PLSD test). **x** shows a statistically significant difference from e18.5 and **y** shows a statistically significant difference from PND0. E–P. *In situ* hybridizations for Esr1 (E, H), Esr2 (F, I, K), and Gper (G, J, L) in PND1 (E–G), PND6 (H-K) and PND2 (L-P) control ovaries show lower Esr1 expression in the ovary than in the oviduct epithelium (Ovd), higher expression of Esr2 in granulosa cells with follicle growth, and expression of Gper in the oocytes and granulosa cells. Inset in F shows a higher magnification of a group of follicles boxed in F. K shows a higher magnification of a primary (Iary and a secondary (IIary) follicles boxed in I. Inset in G shows another section of the ovary containing the oviduct. A comparison of *Gper* mRNA profile by *in situ* hybridization (L) with Esr2 (N, red) and Ybx2 (M, cytoplasmic, green) (and merged pictures O) by immunofluorescence revealed co-expression of both receptors in oocytes at the time of treatment. P shows hybridization with *Gper* sense probe. Scale bar: 100 µm except in insert in F and K (50 µm). Q-T. Quantitative RT-PCR for *Esr1* (Q), *Esr2* (R), *Gper* (S) and *Nr1i2* (T) using ovarian samples of controls (white bars) and animals treated with 10 µg E2 at PND0 (2 h after injection) and PND1 (black bars) shows E2 impairment of post-natal *Gper* up-regulation. Each bar represents mean ± SEM of the fold-change in target gene expression relative to a Snx17 reference gene and calibrator sample. Each point represents mRNAs from 3 to 6 pools of 6–16 and 10–26 ovaries, respectively. *p<0.05 (ANOVA, followed by PLSD test).

Regulation of expression of the well known-nuclear estrogen receptors, *i.e. Esr1, Esr2,* of the membranous estrogen receptor *Gper* and of the nuclear receptor *Nr1i2,* which can bind estrogens and whose expression is regulated at birth in mouse ovary [36], [37] was assessed around the time of treatment by quantitative PCR (Fig. 2A–D). The expression of *Esr1* mRNA significantly decreased between e18.5 and PND12 (Fig. 2A), while expression of *Esr2* mRNA was low at e18.5 and significantly increased from birth to PND12 (Fig. 2B). *Gper* and *Nr1i2* mRNA had similar age-associated expression profiles (Fig. 2C–D).

We investigated which ovarian cell types expressed estrogen receptors around the time of treatment and in later development in ovarian sections using *in situ* hybridization (Fig. 2 E–P, M) and immunofluorescence (Fig. 2 M–O). From PND1 on, we observed a strong *Esr1* mRNA signal in the epithelium of the differentiating oviduct (Ovd in Fig. 2E) and a close-to background signal in the ovary (Fig. 2E, H and Figure S1). *Esr2* mRNA was expressed in the epithelium of the differentiating oviduct as well as in the ovary (Fig. 2F) and its expression increased in granulosa cells with follicle growth at PND6 (Fig. 2 F, I and K). *Gper* mRNA expression pattern was very similar to that of *Esr2* (Fig. 2 G, J and L). The comparison of *Gper* mRNA and Esr2 protein labeling with the oocyte marker Ybx2 showed that oocytes expressed both receptors (Fig. 2 L–O). These data suggest that some Esr1, and to a greater extent Esr2 and Gper, were expressed in the oocytes and (pre-)granulosa cells at the time of estrogen injections.

Since estrogen receptors themselves have been described as direct target of estrogens, we measured the profile of expression of their transcripts in response to a 10 µg–E2 exposure (Fig. 2 N–Q). Quantitative PCR at PND0, between 2 and 4 h after injection, and at PND1 was carried out to investigate the early transcriptomic response of the ovary. *Esr1* had a stable expression between PND0 and PND1 and treatment did not affect its expression (Fig. 2N). Similarly, *Esr2* had a stable expression in the control group between PND0 and PND1 and, although E2 treatment tended to increase the expression between PND0 and PND1, this did not reach statistical significance (Fig. 2O). Expression levels of *Gper* significantly increased between PND0 and PND1 in the control group but remained unchanged in the E2-treated group (Fig. 2P). A similar post-natal increase in *Nr1i2* mRNA expression was found in control ovaries but not observed in E2-treated ovaries (Fig. 2Q).

### Modifications of Ovarian Metabolic Capabilities in Response to E2

**Figure 3:**
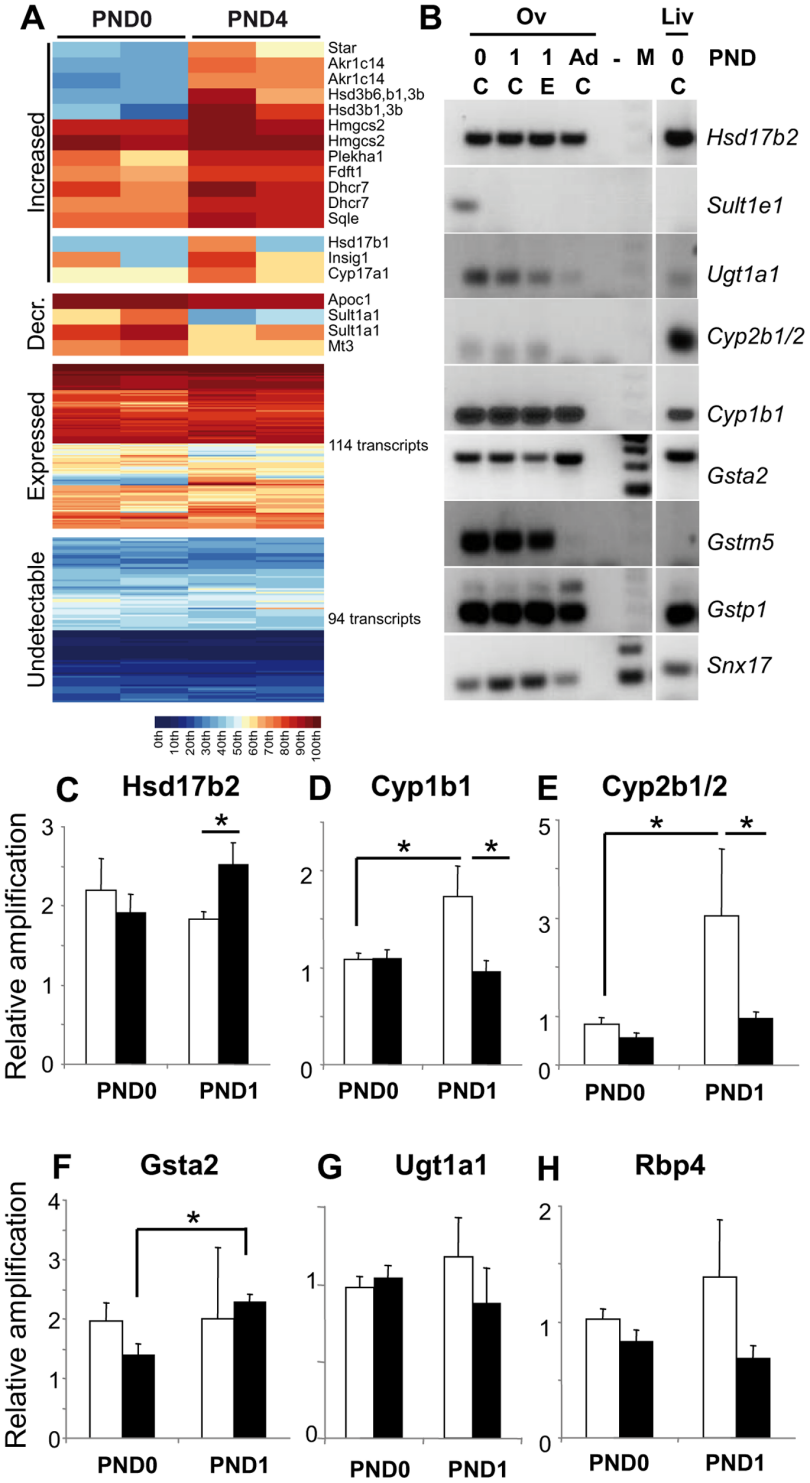
Ovarian reaction to acute estrogen exposure. A. A false-color heatmap shows cases of increasing, decreasing, detectable (but without differential expression) and undetectable transcript signal intensities across the replicates for different total ovary samples at the time points given (top). Each line corresponds to a probe set and each column to a sample replicate. A color scale is shown for signal intensity percentiles (bottom). Gene symbols and numbers of transcripts are shown at the right. B. Conventional RT-PCR screening of the expression of various enzymes involved in E2 metabolism in PND0, control (C) and E2-treated (E) PND1 and adult female ovaries, and PND0 livers reveals changes in expression of *Ugt1a1*, *Gsta2*, *Gstm5* between newborn and adult ovaries, stable expression of *Hsd17b2*, *Cyp1b1*, *Gstp1*, and the faint expression of *Sult1e1* and *Cyp2b1/2* by contrast to control *Snx17* RNAs. C–H. Quantitative RT-PCR for *Hsd17b2* (C), *Cyp1b1* (D), *Cyp2b1/2* (E), *Gsta2* (F), *Ugt1a1* (G) and the *Rbp4* E2 target gene (H) in ovaries of controls (white bars) and animals treated with 10 µg E2 (black bars) at PND0 (2 h after injection) and PND1 shows E2 impairment of post-natal *Gper* up-regulation. Each bar represents mean ± SEM of the fold-change in target gene expression relative to a Snx17 reference gene and calibrator sample. Each point represents mRNAs from 3 to 6 pools of 6–16 and 10–26 ovaries, respectively. *p<0.05 (two-way ANOVA, followed by Tukey test).

To get insights into the expression of enzymes of the E2 metabolic pathway in the post-natal ovary, we explored already published transcriptomic profiles of PND0 compared to PND4 rat ovaries [34]. We restricted our analysis to the 227 probe sets (transcripts) associated with the GO term annotation “steroid metabolic process” (GO:0008202). Out of the 133 probe sets with detectable intensity signals in either PND0 or PND4, 114 transcripts did not display differential expression. However, we found 12 to be associated with a significantly increased expression pattern from PND0 to PND 4, and four to be associated with a significantly reduced expression pattern (Fig. 3A). Enzymes involved in E2 biosynthesis increased after birth (statistically significant differences in mRNA expression for *Star* and *Hsd3b1*, but not significant for *Cyp17a1 and Hsd17b1*). This may well correlate with the very first steps of theca-interstitial tissue differentiation. In contrast, mRNAs encoding enzymes involved in E2 transformation such as *Sult1a1* and, to a lesser extent, *Hsd17b2*, and *Cyp1b1* decreased in prevalence between PND0 and PND4 (Fig. 3A). Because the microarrays used in this study covered approximately half of the known protein-coding genes, data were partial. We thus selected enzymes of each branch of E2 detoxification pathway to compare by quantitative PCR their expression in PND0 and PND1 neonatal and adult ovaries as well as in PND0 livers (Fig. 3B). The mRNA levels of *Hsd17b2, Cyp1b1, Cyp2b1/2, Gsta2* and *Gstp1* were globally constant over time. By contrast, *Sult1e1, Ugt1a1* and *Gstm5* were preferentially expressed in the neonatal ovary and merely absent in the adult organ. Of note, *Ugt1a1* and *Gstm5* were preferentially expressed in the ovary while *Cyp2b1/2* was preferentially expressed in the liver, thus highlighting different pathways of detoxification in each organ. Altogether, these results suggest that the ovary possesses the machinery to locally metabolize E2.

We used quantitative PCR to investigate variation in the expression of selected estrogen metabolism enzymes in response to E2 exposure (Fig. 3 C–F). The expression of *Hsd17b2* was similar in the control and treated groups at PND0. Whereas it remained constant at PND1 in the control group, E2 treatment induced a significant increase in expression in the treated group (Fig. 3C). *Cyp1b1* mRNA levels did not vary 2 h after the E2 injection (Fig. 3D). However, whereas *Cyp1b1* mRNA levels increased significantly between PND0 and PND1 in the control group, they remained unchanged in the treated group (Fig. 3D). The expression of *Cyp2b1/2*, which was slightly greater in the control group than in the treated group at PND0, dramatically increased between PND0 and PND1 in control group but did not significantly change in the E2-treated group (Fig. 3E). Expression of *Gsta2* did not vary in the control group, but significantly increased in the treated group between PND0 and PND1 (Fig. 3F). *Ugt1a1* mRNA profile was not modified with either time or treatment (Fig. 3G). Rbp4, which has previously been shown to be a target of E2 in the fetal and juvenile rat ovaries [38], followed the same trend as *Cyp1b1* and *Cyp2b1/2*, albeit not significantly (Fig. 3H).

### Estrogen Dose-dependent Depletion of Oocyte Pool

**Figure 4:**
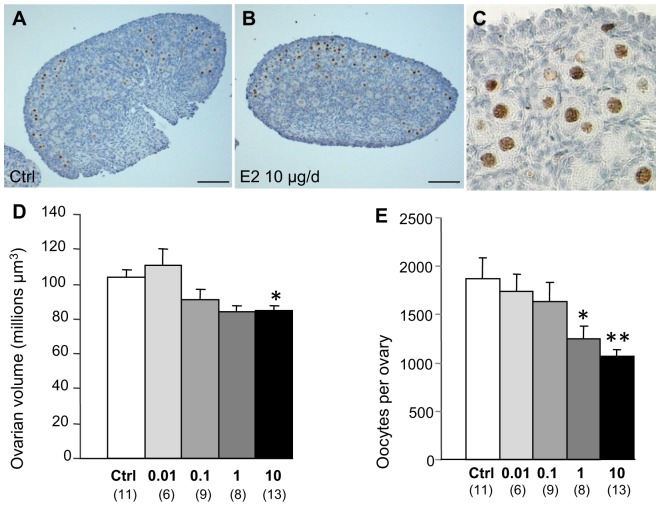
Estrogen dose-dependent depletion of oocytes. A–C. Immunolabeling of TRA98 in oocytes of control animals at PND3 (A) and females treated daily with 10 µg/d E2 between PND0 and PND2 (B). Higher magnification of TRA98 labeling shows oocyte-specific expression (C). Scale bar = 100 µm. D–E. Stereological estimation of ovarian volume (D) and oocyte numbers per ovary (E) at PND3 in controls, and as a function of E2 dose, show a dose-dependent decrease of both parameters. Each bar represents means ± SEM. Number of ovaries are indicated in brackets on the abscissa. *p<0.01; **p<0.001 (ANOVA followed by Fisher test).

Repeated 10 µg E2 treatment is known to effect the volume of infantile [22] and pre-pubertal ovaries [39]. Since sections of PND3 ovaries of the 10 µg/d group were smallest compared to control ones at the histological level (Fig. 4A–C), we performed stereological measurements of ovarian volumes for each dose group (10, 1, 0.1 and 0.01 µg/d) after the end of the treatment at PND3 (Fig. 4D). Ovarian volume followed a dose-dependent decrease after E2 treatment but this was only statistically significant for the 10 µg/d dose (Fig. 4D). Because this modification was rapid, we chose to evaluate the total number of oocytes instead of categorizing naked/primordial/primary follicles by morphological criteria using oocyte nucleus labeling with GENA [40] (Fig. 4E). Oocyte population decreased as a function of the dose of injected E2: at doses of 1 µg/d and above, the population size was significantly different to that of control individuals. These data suggested that neonatal E2 supplementation depletes the oocyte pool in female rats. We used the 10 µg dose for further investigations as this dose had effective effects on both ovarian volume and oocyte number.

**Figure 5:**
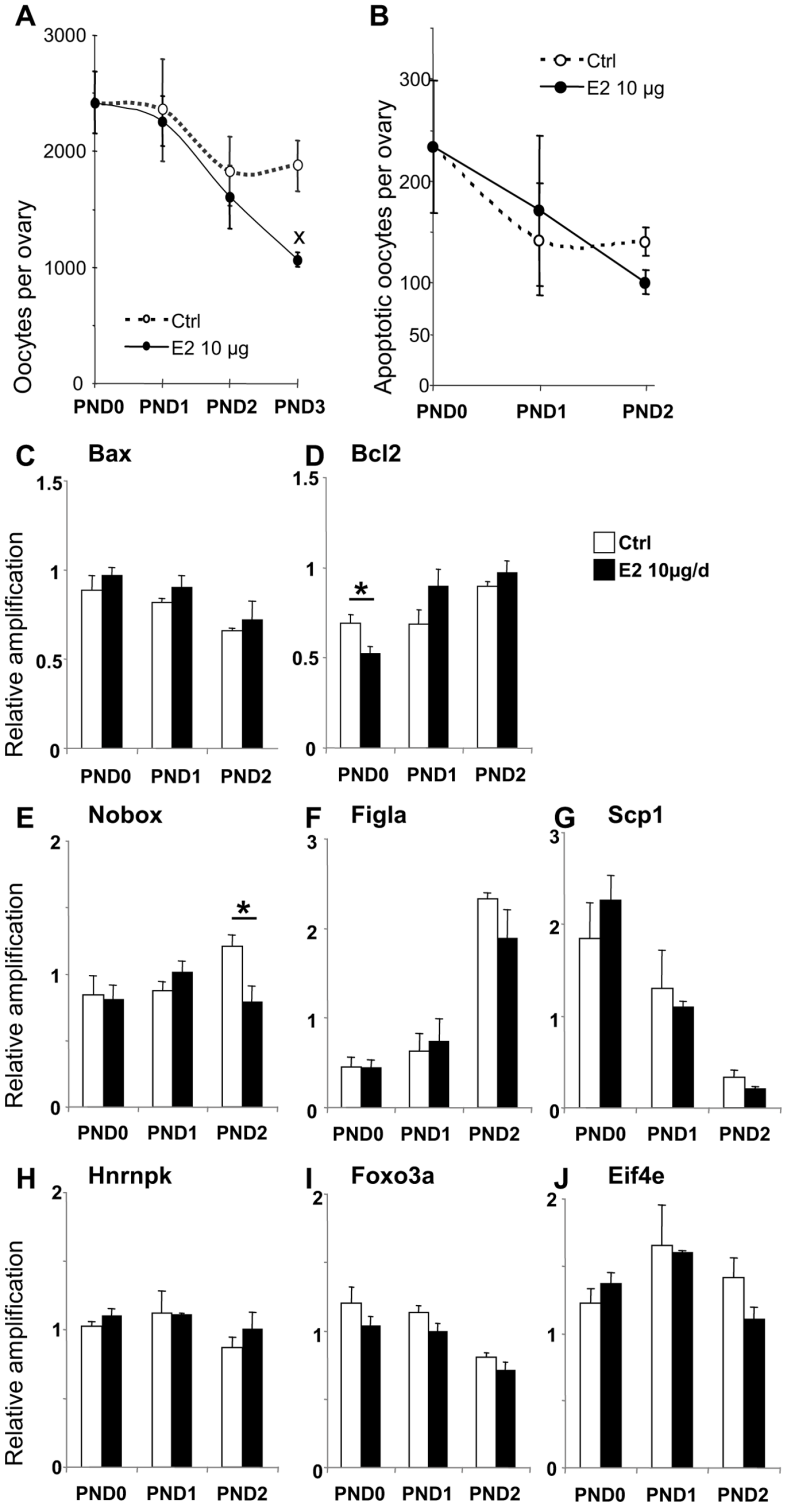
Characterization of oocyte depletion. A. Time-course showing the decline in oocyte number per ovary in control (white points and dotted lines) and 10 µg E2-treated ovaries (black points and continuous lines) from PND0 to PND3. Data are expressed as mean ± SEM of 5–14 ovaries. *p<0.01 *vs.* age-matched controls (ANOVA followed by PLSD test). B. Time-course of apoptotic oocyte number per ovary in control (white points and dotted lines) and 10 µg E2-treated ovaries (black points and continuous lines) from PND0 to PND2. Data are expressed as mean ± SEM of 4–8 ovaries. C–D. Quantitative RT-PCR for *Bax* (C), *Bcl2* (D), *Nobox* (E), *Figla* (F), *Scp1* (G), *Hnrnpk* (H), *Foxo3a* (I) and *Eif4e* (J) in ovarian samples of controls (white bars) and animals treated with 10 µg E2 (black bars) at PND0 (2 h after injection) and PND1 shows transient down-regulation of *Bcl2* mRNA at PND0 and the absence of *Nobox* mRNA up-regulation between PND1 and PND2 in E2-treated ovaries. Each bar represents mean ± SEM of the fold-change in target gene expression relative to a Snx17 reference gene and calibrator sample). Data from 3–6 pools of 6–14 ovaries. *p<0.05 *vs.* age-matched control (two-way ANOVA, followed by t-test).

We analyzed the time-course of oocyte depletion after a 10 µg/d E2 treatment (Fig. 5A). Oocyte numbers decreased with a similar slope between PND0 and PND2 in control and treated groups and oocyte depletion extended for one more day in E2-treated females (Fig. 5A). Since oocyte apoptosis is a physiological process that characterizes follicle formation [12], [14], we quantified apoptotic oocytes after TUNEL assays between PND0 and PND2 in control and E2-treated females (Fig. 5B). Both groups of females displayed a similar post-natal decline in apoptotic oocyte number from PND0 to PND2 and we could not detect any difference between control and treated ovaries (Fig. 5B). In agreement, the mRNA levels of the pro-apoptotic *Bax* gene (measured by quantitative PCR) were stable in both control and treated ovaries between PND0 and PND2 (Fig. 5C). By contrast, the mRNA levels of the anti-apoptotic *Bcl2* gene were steady stable in control ovaries while lower to control ones 4 h after the first injection and then increasing in E2-treated ovaries (Fig. 5D).

We investigated the expression of genes proposed to be involved in follicle formation (*Figla*, *Hnrnpk*, *Foxo3a* and *Nobox*) and meiosis progression (*Eif4e*, *Scp1*) [41]–[47]. To detect early changes triggered by the E2 treatment, quantitative PCR were performed before a decrease in oocyte number was detected (at PND0, PND1 and PND2). E2 treatment only affected the profile of *Nobox* expression at PND2 (Fig. 5E–J): *Nobox* mRNA expression increased between PND1 and PND2 in control ovaries but not in E2-treated ones. This suggested that, apart from *Nobox*, genes classically involved in follicle formation may not have a role in E2-induced oocyte depletion.

### Abnormalities of Follicle Formation in Response to E2 Exposure

**Figure 6:**
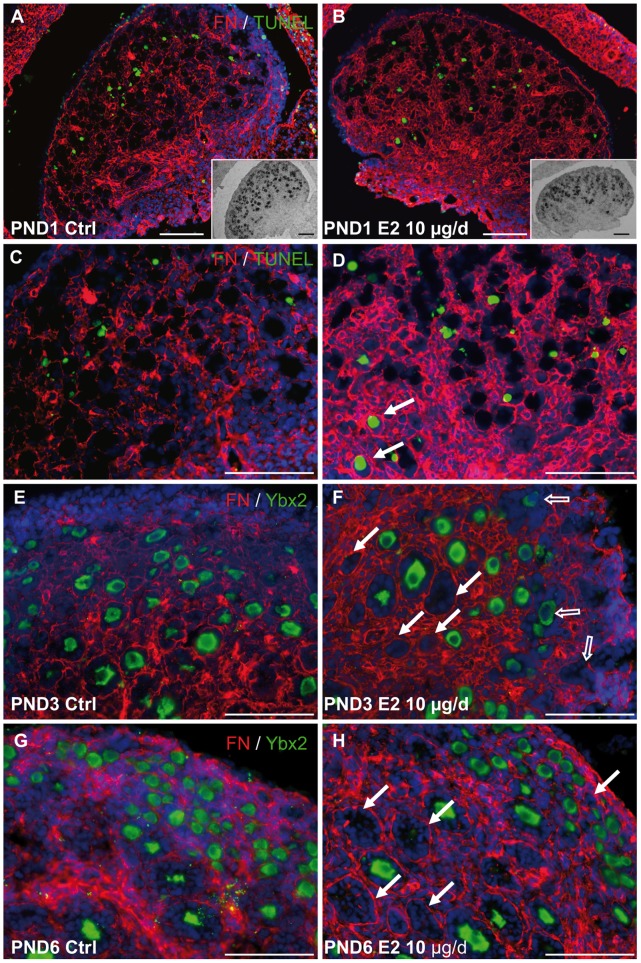
Abnormalities of follicle formation. A–D. Comparison of apoptosis using TUNEL (green), fibronectin (red) and nuclei (DAPI, blue) in control (A, C) or 10 µg-E2 treated (B, D) ovaries at PND1 highlights physiological oocyte apoptosis in the areas of ovarian cords remodeling in control ovaries, and additional oocyte apoptosis in already formed follicles in E2-treated ovaries (arrows). Insets in A–B show *in situ* hybridization against *Ybx2* performed on the same section. C–D are higher magnifications of A–B. Scale bar: 100 µm. E–H. Comparison of oocytes labeled with Ybx2 (green) and basement membrane labeled with fibronectin (red) and nuclei (DAPI, blue) in PND3 (E–F) and PND6 (G–H) control (E, G) and 10 µg E2-treated ovaries (F, H) show incomplete basement membrane remodeling at PND3 in E2-treated ovaries (open arrows) and empty follicles at PND3 and PND6 (arrows). Pictures are representative from 4–6 ovaries. Scale bar: 100 µm.

The wave of physiological oocyte programmed cell death progresses from core areas to the periphery of the ovary in rodents [48]. Degenerating oocytes are located in areas of new basement membrane deposition which delineate the emerging follicular units [11]. We investigated the topographical pattern of oocyte degeneration by comparing TUNEL-positive cells and fibronectin-labeled extra-cellular matrix at PND1 (Fig. 6A–D). Whereas typical apoptotic oocytes were found close to basal membrane remodeling in both control and E2-treated ovaries, numerous degenerating oocytes, already enclosed in follicles, were observed in the core of the E2-treated ovaries (Fig. 6D).

Because MOFs are a hallmark of neonatal estrogenization, we investigated cord fragmentation by analyzing fibronectin-labeled basal membrane deposition around newly formed follicular units (Fig. 6 E–H). Ovarian cord fragmentation was nearly complete in control animals at PND3 (Fig. 6E) but follicle formation anomalies, such as epithelial structures devoid of oocyte and incomplete cord fragmentation, were observed in 10 µg-treated animals (Fig. 6F). At PND6, an age at which follicle formation is known to be completed (Fig. 6G), many abnormalities (such as follicular units devoid of oocytes) remained in E2-treated ovaries (Fig. 6H).

## Discussion

Our results provide direct evidence that the newborn female rat rapidly acquires the capability to partially protect itself against repeated E2 injections by decreasing E2 in the plasma. This is associated with a change in the expression profiles of several hepatic detoxification enzymes involved in its own metabolism. Interestingly, the ovary itself expresses enzymes for E2 metabolism and modifies their expression in response to E2. We show that neonatal E2 exposure can directly affect oocyte survival in a dose-dependent manner in the rat. Oocyte depletion is time-dependent for the highest dose tested and affects already formed follicles in addition to areas of ovarian cord remodeling. The data highlights a dramatic species difference as experimentally-increased E2 levels promote oocyte survival in the mouse.

### Whole Organism Response to E2

In our control female pups, E2 levels decreased from 90+/−3 pg/mL at PND0 to 24+/−11 pg/mL at PND3 and fell to 2+/−2 pg/mL at PND6. The 0.01, 0.1, 1 and 10 µg/d doses raised the levels up to 80+/−17, 233+/−199, 618+/−34 and 3685+/−968 pg/mL at PND1, respectively. By comparison, mean E2 levels in ovarian venous blood during pregnancy have been described to plateau at levels between 120+/−19 to 199+/−55 pg/mL between e3 and e17, which are close to estrus levels (161+/−28 pg/mL), then raise up to 445+/−115 pg/mL at e21, 348+/−74 at e22, and peak at 628+/−210 pg/mL at parturition [49]. Studies on xeno-estrogen effects on the ovary and hypothalamus classically use a 10 µg/d E2 dose as a positive control [22], [27], [50]–[54]. By comparing the dynamics of E2 plasma levels in controls and animals treated with different doses of E2, we showed that doses from 0.1 µ/d tend to increase circulating E2 levels above control levels at PND1. This became statistically significant for the 10 µg/d dose, which raised circulating levels by up to 15 times that of the physiological levels at PND1. Thereafter, it was not possible to maintain PND0 E2 levels by E2 injection with a given dose because plasma E2 levels decreased from PND1 to PND3 despite repeated injections in the different treatment groups. This indicated that newborns progressively acquire the capability to clear E2 form the organism. A return to levels similar to that of controls was reached at PND6 for all dose groups but not at PND3, *ie* only a few days after termination of the treatment. The only dose where circulating levels were found to be higher than control levels during the whole course of the treatment was 10 µg/d. Consequently, the effective dose at the systemic level is 10 µg/d. In addition, although studies on neonatal estrogen exposure use either E2 or esters of E2 that display different half lives in the organism [55], the effective dose is typically 1–10 µg/d at the hypothalamus level [22], [27], [50]–[54]. By addressing dose effects, we show here that a 10 µg/d is also the effective dose in the ovary.

Altogether, our results on hormone profiling and mRNA quantification after E2 injections suggest that the newborn rat acquires the complex capability to metabolize E2 according to fine dynamics in the days following birth. Estradiol metabolism is carried out by enzymes that convert E2 to a biologically less active estrogen (E1) and by phase-I and II enzymes that catalyze oxidation and conjugations to permit their rapid excretion by the kidney (Fig. 1A). The decrease of circulating E2 in controls suggests that mother-derived E2 is cleared by the newborn, insofar as the newborn itself does not synthesize E2. At birth, the rat ovary is not able to produce E2 [56] until the granulosa cells of the first wave of follicular growth typically express *Cyp19a1*. This typical expression is associated with the peak levels of E2 during the infantile period [5], [6]. By PND2, even after repeated injections of the highest dose, circulating E2 levels decreased concomitantly with a sharp increase of E1. This suggests that newborn females can convert excessive E2 into E1, which is supported by Hsd17b2 activity. Subsequent conversion of E2 and E1 into hydroxyl-metabolites is handled by typical phase I enzymes of the Cyp family, *ie* Cyp1a2, 2b1/2, 2c6, 2c11 and the 3a family [57]–[59]. Phase II estrogen metabolism includes sulfatation, glucuronidation, *O*-methylation and glutathione-conjugation, which are catalyzed by sulfo- (Sult), UDP-glucuronosyl- (Ugt), catechol-*O*-methyl- (COMT) and Glutathion-S-Transferases (Gst), respectively. Sulfates and glucuronides are the most abundant circulating estrogen conjugates [60]. While glucuronides are primarily excretion forms, sulfate derivatives have also been described as storage forms [60], [61]. The absence of statistical changes in circulating levels of sulfated estrogens, excepted for E1S at PND3, could reflect either the absence of increased sulfatation process (and possibly the absence of regulation of expression and/or activity of enzymes in charge of this process), or an increase of the excretion of sulfate-conjugated estrogens, or both.

We were not able to measure the hydroxyl- and glucuronide-conjugated metabolites of E2 and E1 because of their instability and the low quantity of plasma that can be recovered in each newborn. We therefore investigated hepatic regulation of the mRNA expression of selected relevant enzymes of the E2 metabolic pathway. In agreement with the well-known post-natal maturation of the hepatic endowment of enzymes of lipid metabolism [7], [62], [63], we found up-regulation of *Hsd17b2, Cyp2b1/2* and *Gsta2* and down-regulation of *Cyp1b1* mRNA levels between PND0 and 1. In contrast, transcripts encoding Ugt1a1 (which contribute to glucuronide conjugation together with Ugt1a3, 1a8, 1a9, 1a10 and 2b7 [64], [65], [66]), were not modified between PND0 and PND1. Despite its major protecting role against E2 effects in male tissues, and the clear reproductive phenotype in females lacking its expression [67], [68], it was not possible to investigate Sult1e1 regulation of expression as it was expressed at levels below the qPCR threshold.

Our mRNA quantifications in the liver suggest that E2 itself, at least at the highest dose, inhibited the physiological up-regulation of hepatic *Hsd17b2* and *Cyp2b1/2* mRNA, which encode two key enzymes of the E2 detoxification pathway. This could indicate that the level of enzymes in the newborn liver is sufficient for E2 conversion. Alternatively, E2 treatment could counter the physiological acquisition of the newborn liver to convert E2 into less active metabolites. By contrast, E2 treatment had no effect on physiological neonatal *Cyp1b1* down-regulation and *Gsta2* up-regulation of expression in the liver. Cyp1b1 response to E2 exposure seems to be organ-specific since its expression is either inducible by E2 and deoxymiroestrol (a strong phyto-estrogen in mouse hepatocyte primary culture [69]) or down-regulated by estrogenic compounds in peripheral organs such as rat and mouse testis and the reproductive tract of developing female rats [38], [70], [71]. This may also be the case for Gst enzymes as genistein increases Gsta2 mRNA levels and decreases Gstp1 mRNA levels without affecting general GST activity in adult males, and deoxymiroestrol increases Gsta2 expression in adult mice hepatocyte primary culture [69], [72]. Finally, hepatic *Ugt1a1* mRNA levels were not modified by E2. This would suggest that E2 treatment has no influence on the expression of this enzyme at this age, unlike in adult mouse livers where its expression is inducible [73]. Nevertheless, the absence of effect of E2 on *Ugt1a1* and *Gsta2* mRNA neonatal profiles does not preclude a modification of expression of other Gst and Ugt, or a modification of enzyme activity. By targeting selected enzymes of the metabolizing pathway, E2 treatment may well favor one or more metabolic branches over others. Collectively, our data led us to hypothesize that by reproducing the maternal E2 environment, high levels of E2 levels would force the liver to remain in a relative immature state. Therefore, as suggested in the ovary, lifting the maternal E2 environment at birth could enable maturation of the newborn liver and development of its own detoxification machinery.

### Arguments in Favor of the Existence of an Ovarian Local Metabolic Response to E2 Exposure

Neonatal estrogen exposure classically induce immediate transcriptomic effects and long-lasting physio-pathological effects in reproductive tissues such as the testis, ovary, uterus, oviduct and hypothalamus, but also in other tissues including bone, kidney and liver [74]. The search of E2 target genes is the main outcome that is commonly investigated at the transcriptomic level. Amongst target genes, E2 induces modifications of the detoxification machinery in the rat adult female liver, which is the major drug-metabolizing organ of the body [75], [76], but also in reproductive organs such as uterus, testis and fetal and juvenile ovaries [38], [77], [78]. In agreement, our results suggest that local mechanisms may exist in the neonatal ovary as well. Indeed, the ovary responds to E2 exposure by modifying the expression of several E2 metabolism pathway enzymes. Although *Hsd17b2* mRNA was not thought to be expressed in the immature and mature rat ovary [79], we found an up-regulation above basal levels in response to a 10 µg E2 injection that may increase the capability of the ovary to transform E2 into its less active E1 metabolite [80]. On the opposite, the PND0 to PND1 up-regulation of *Cyp1b1* and *Cyp2b1/2* mRNA levels was not observed in response to a 10 µg E2 injection, suggesting a possible decreased capability to locally transform circulating E2 and E1 into excretable hydroxyl- and methoxy-estrogen metabolites. We propose that, as in the liver, E2 treatment could also counter local ovarian acquisition of the machinery involved in its own metabolism, as illustrated by a similar inhibition of post-natal *Cyp2b1/2* mRNA up-regulation. The local ovarian mRNA up-regulation of *Hsd17b2* may locally rescue, at least in part, the absence of up-regulation in the liver.

An auto-sensory mechanism adjusting the expression of detoxification enzymes would involve local signaling via estrogen receptors. In agreement with previous reports in the rat [81], [82], we confirm lower expression of Esr1 in the neonatal rat ovary than in the oviduct, expression of Esr2 in (pre-)granulosa cells which increases with follicular growth, and low expression of Esr2 in the cytoplasm of oocytes at birth. We also extend the knowledge of Gper expression pattern in the rat to the neonatal period in granulosa cells, oocytes, and surface epithelial cells of the ovary and the oviduct, consistent with the pattern reported in the hamster [19], [83]. Altogether, the neonatal rat ovary has the machinery to respond to E2. In contrast to other organs or developmental periods, we were not able to show any modification of either Esr1 or Esr2 expression associated with the 10 µg/d E2 treatment. *Gper* mRNA, which has not been described as a classical E2 target gene to our knowledge, did not display a physiological increase between PND0 and PND1 in response to E2. In the hamster, Gper was proposed to mediate E2 action on follicle formation, as *in vitro* ablation with small interfering RNAs markedly suppressed estrogen-stimulated primordial follicle formation [19]. E2-disruption of post-natal Gper increase may suggest a similar role of Gper in follicle formation in the rat. The same trend of response to E2, albeit not statistically significant, was found for *Nr1i2* expression in agreement with mouse ovarian data). Interestingly, the sharp increase in *Nr1i2* expression in the prenatal mouse ovary followed by a post-natal decrease at PND3, that we also found in the rat ovary, was proposed to reflect a role for this receptor in protecting the feto-maternal system from the toxic effect of endogenous steroids and foreign substrates [36]. Unfortunately, *Nr1i2* mRNA levels were below our *in situ* hybridization detection levels so we were unable to identify the cells that express Nr1i2. The low expression level of Nr1i2, together with the absence of modification to its expression by E2, reduces the likelihood of its involvement in the response to E2 high levels.

### Estradiol and Oocyte Survival

Although the 10 µg/d E2 treatment induced a local modification in the expression of genes encoding metabolism enzymes, we showed that E2 exposure at the time of follicle formation induced a dose-dependent ovotoxicity. The effect of E2 on the neonatal ovary is often evaluated at the follicle level and previous studies have shown that neonatal E2 exposure induces a moderate reduction in the rate of primordial follicle assembly, a lower level of initial primordial-to-primary follicle transition [16] and fewer layered follicles at PND6 [84]. Similarly, neonatal exposure to BPA reduces the pool of primordial follicles in the rat ovary [28]. Nevertheless, there may be multiple effects of E2 on follicle assembly and/or primordial follicle activation. By focusing on the total oocyte number, we showed a rapid effect of E2 on oocyte survival, irrespective of the structure in which it is included. Further studies are necessary to identify the fine mechanisms by which E2 affects oocyte survival. However, our results contrast with those reported in mice in which a similar range of EB doses (0.1 to 1 µg/mouse) decrease the number of apoptotic germ cells at PND5 [85]–[87]. In other in vivo studies in mice, 10 or 20 µg/day of ethinyl estradiol did not modify whereas 100 µg/day increased the number of oocyte per section [18], [88], [89], highlighting the importance of the dose. In agreement, studies in hamsters revealed that doses of E2 within the physiological range reduced apoptosis and stimulated the formation of follicles, and that doses above a threshold level had no effect on primordial follicle formation but significantly up-regulated oocyte apoptosis [20]. We show a dose-effect of E2 on oocyte depletion in the rat but no biphasic response.

There may be several reasons for species-differences in ovarian E2 sensitivity. Distinct estrogen receptivity at the time of follicle assembly are unlikely because of the highly conserved expression profiles of the different estrogen receptors, especially between these rodent species [82], [83], [90]. Subtle species differences between rat and mice have been reported in the fine mechanisms governing the programmed cell death of oocytes at birth. Neonatal oocyte attrition in mice involves apoptosis and autophagy mechanisms in different cells [91], but in rats, dying oocytes can share several features of both processes [14]. Alternatively, the xeno-estrogen Bisphenol A was shown to hamper meiotic progression, resulting in oocyte apoptosis in mice and humans [92], [93]. This indicates that E2 could interfere with the timing of meiosis arrest during follicle histogenesis and ultimately induce oocyte death. Our search for meiosis or oocyte-specific marker profiles in response to E2 treatment showed that only *Nobox* expression was modified in ovaries of E2-treated females. Indeed, *Nobox* mRNA did not display the up-regulation observed between PND0-1 and PND2 in control ovaries. This increase in controls may reflect the expression of *Nobox* in oocytes of primordial and growing follicles and thus the maturation of the organ. In E2-treated ovaries, the absence of increased *Nobox* expression between PND1 and PND2 could indicate a default in the primordial-to-primary follicle transition or a poor growth of the first wave of follicles. However, this is countered by the absence of modification of expression in several other oocyte-specific genes. Since mice lacking *Nobox* display accelerated post-natal oocyte loss associated with a decrease in the expression of numerous oocyte-specific genes [44], [94], the absence of increase of *Nobox* could involve this pathway in the mechanism by which oocytes disappear after E2 exposure. This alternative mechanism would correlate well with the delayed decrease in oocyte numbers between PND2 and PND3. Nevertheless, although we were not able to detect any changes of the number of TUNEL-positive apoptotic oocytes, or global expression of apoptosis-related genes, we showed difference in areas and structures where apoptotic oocytes were included. This would suggest that E2 induces abnormal apoptosis in already formed follicles rather than increasing physiological process. Abnormal apoptosis of oocytes in already formed follicles correlates well with the observation of numerous empty follicles at PND3 and PND6. At the organ level, the number of oocytes involved in cell death is very low; this would explain the absence of variation of several selected oocyte markers. Further work is needed to define the fine mechanisms of oocyte disappearance after E2 exposure.

### Estradiol and Abnormalities of Follicle Formation?

MOFs are a hallmark of several mammalian species but their etiology is unknown [95]. Studies using E2 and E2-mimetics in mice suggest that a decrease in oocyte apoptosis and cyst breakdown ultimately results in MOFs [18], [86]. By contrast in the rat, massive oocyte depletion following fetal irradiation is associated with an increased incidence of MOFs [96]. Consequently, the etiology of MOFs in rat and mouse may well differ. We show here that E2 exposure is associated with neonatal oocyte depletion but we did not observe MOFs in older ovaries (at PND6, PND21 and in adults, data not shown). This is in agreement with studies in rats exposed to DES and BPA where MOFs were not reported, or disappeared before puberty [27], [28], [51], [84]. The timing of oocyte depletion, between PND2 and PND3 and the presence of apoptotic oocytes in already formed follicles at PND1 suggest that the absence of MOFs may be explained by a depletion of oocytes after follicle formation.

It is well established that the moment of germ cell depletion will determine the subsequent ovarian phenotype. Irradiation-induced fetal depletion of germ cells in the rat results in streak gonads, whereas PND5-oocyte depletion leads to empty-primary follicles evolving into cord-like structures where granulosa cells trans-differentiate into Sertoli-like cells [97]–[99]. Neonatal exposure to E2 in the ovaries leads to a phenotype that is more closely related with neonatal irradiation, with a decreased oocyte pool. We here show that neonatal E2 exposure induced oocyte depletion in already formed follicles. Although further investigations on the fate of pathological empty follicles are needed, we cannot exclude the possibility that neonatal E2 exposure may also target the first wave of follicle growth and follicle dynamics at the end of the first week. Indeed, studies focusing on follicle dynamics in infantile rat ovaries after neonatal E2 exposure have produced contradictory results: some indicate that E2 increases primordial-to-primary follicle transition *in vitro* and *in vivo* at PND8 while others report a decrease in the number of layered follicles at PND6 [16], [28], [84]. However, by focusing on total oocyte numbers, our study shows that E2 affected oocyte survival independently of the structure in which oocytes are enclosed, in addition to its possible impact on follicle dynamics.

Our study shows that a neonatal exposure to high E2 levels interferes with the maturation of expression of key detoxification tools in the liver and ovaries. In the rat ovary, E2 is ovotoxic in a dose-dependent manner. A special care must be taken when assigning the E2 reference dose in future studies to enable accurate comparisons with environmental contaminants displaying estrogenic activities.

## Supporting Information

Figure S1 **Expression of Esr1 in the neonatal rat ovary.** A–F. *In situ* hybridizations for Esr1 (A, C, E), and Esr1 sense probe (B, D, F) in PND2 ovaries show a high level of expression of Esr1 in the epithelium of the differentiating oviduct (compare C to D), and in some cells of the mesenchyme of the oviduct (open arrow in C) and a close-to-background expression in oocytes (arrows in E, compare to F) and an expression in the ovarian surface epithelium (E, compare with F). G Double staining of E with Ybx2 by immunofluorescence to labeled oocytes (arrows, red). Nuclei are counterstained with DAPI (blue). H Merged pictures of immunofluorescence for Esr1 (red), Ybx2 (green) and cell nuclei (blue) in a PND1 ovary shows a high expression of Esr1 in cells of the oviduct mesenchyme (open arrow), an expression in epithelial cells of the ovarian surface and follicles (arrowheads) and in oocytes (arrow). Scale bars: 100 µm.
(TIF)

Table S1 **GC-MS serum analytical control validation.** LLOQ: low limit of quantification; QC: quality control.
(DOC)
