# Correction: Systemic Compensatory Response to Neonatal Estradiol Exposure Does Not Prevent Depletion of the Oocyte Pool in the Rat

**DOI:** 10.1371/annotation/2cd33d2f-684d-44ae-9888-bcac7d69c1e9

**Published:** 2014-01-02

**Authors:** Clémentine Chalmey, Franck Giton, Frédéric Chalmel, Jean Fiet, Bernard Jégou, Séverine Mazaud-Guittot

The name of the second author is spelled incorrectly. The correct name is: Frank Giton.

