# Supplementary figures and images for: Systemic Compensatory Response to Neonatal Estradiol Exposure Does Not Prevent Depletion of the Oocyte Pool in the Rat
Source: PLoS One. 2013 Dec 16;8(12):e82175. doi: 10.1371/journal.pone.0082175 (PMC3864944; doi:10.1371/journal.pone.0082175)

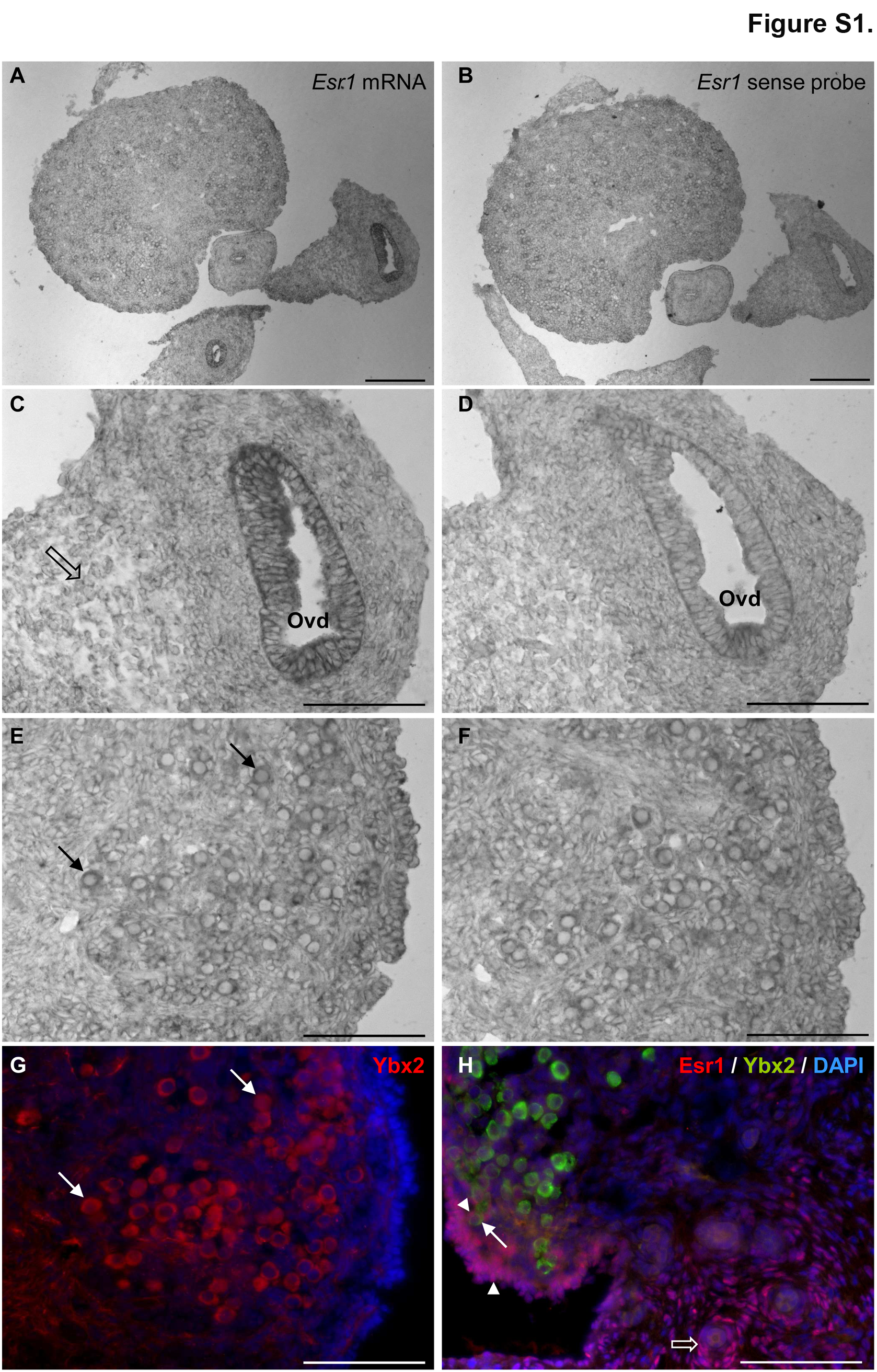

Supplement: Figure S1 — Expression of Esr1 in the neonatal rat ovary. A–F. In situ hybridizations for Esr1 (A, C, E), and Esr1 sense probe (B, D, F) in PND2 ovaries show a high level of expression of Esr1 in the epithelium of the differentiating oviduct (compare C to D), and in some cells of the mesenchyme of the oviduct (open arrow in C) and a close-to-background expression in oocytes (arrows in E, compare to F) and an expression in the ovarian surface epithelium (E, compare with F). G Double staining of E with Ybx2 by immunofluorescence to labeled oocytes (arrows, red). Nuclei are counterstained with DAPI (blue). H Merged pictures of immunofluorescence for Esr1 (red), Ybx2 (green) and cell nuclei (blue) in a PND1 ovary shows a high expression of Esr1 in cells of the oviduct mesenchyme (open arrow), an expression in epithelial cells of the ovarian surface and follicles (arrowheads) and in oocytes (arrow). Scale bars: 100 µm. (TIF) [file pone.0082175.s001.tif]
