# Supplementary material for: Systemic Compensatory Response to Neonatal Estradiol Exposure Does Not Prevent Depletion of the Oocyte Pool in the Rat
Source: PLoS One. 2013 Dec 16;8(12):e82175. doi: 10.1371/journal.pone.0082175 (PMC3864944; doi:10.1371/journal.pone.0082175)
Supplement: Table S1 — GC-MS serum analytical control validation. LLOQ: low limit of quantification; QC: quality control. (DOC) [file pone.0082175.s002.doc]

**Table S1.**

| **Accuracy** | **Analytes** | **Target ions analyte / IS**  **(amu)** | **Range of detection**  **(pg/ml)** | **Mean (43 runs)**  ***Intra- & Inter assay CVs (%)*** | | | |
| --- | --- | --- | --- | --- | --- | --- | --- |
|  |  |  |  | **LLOQ** | **Low QC** | **Middle QC** | **High QC** |
| Accuracy for E1, E2, E1-S and E2-S measured by GC-MS in human samples :  -3.9 % - +3.5 %  (n = 6) | E1 | 464 / 468 | 8 - 810 | 7.9 | 14.4 | 77.9 | 198.9 |
|  |  |  |  | *7.4 - 9.5* | *4.3 - 4.7* | *3.1 - 3.3* | *2.6 - 3.0* |
|  | E2 | 660 / 664 | 2 - 405 | 2.1 | 9.8 | 48.0 | 198.3 |
|  |  |  |  | *8.9 - 10.5* | *3.5 - 4.1* | *2.6 - 3.0* | *1.5 - 1.7* |
|  | E1-S | 464 / 468  (after solvolysis) | 50 - 6000 | 49.8 | 500.4 | 1454 | 2992 |
|  |  |  |  | *8.7 - 11.0* | *4.0 - 4.4* | *3.1 - 3.4* | *2.8 - 2.9* |
|  | E2-S | 660 / 664  (after solvolysis) | 10 - 1350 | 10.1 | 101.3 | 299.5 | 599.0 |
|  |  |  |  | *3.9 - 5.4* | *3.0 - 4.2* | *3.0 - 4.1* | *2.8 - 3.2* |
